# Effects of Ecological Environment on Physiological Indicators of Facial Skin in Hainan

**DOI:** 10.1111/jocd.70621

**Published:** 2025-12-18

**Authors:** Jingqiu Fu, Wenxia Huang, Yujia Liang, Rui Wang, Xiaoli Chen, Jiejie Lu, Weiwei Wu

**Affiliations:** ^1^ Department of Dermatology The Fifth People's Hospital of Hainan Province Haikou Hainan People's Republic of China; ^2^ Department of Dermatology Affiliated Dermatology Hospital of Hainan Medical University Haikou Hainan People's Republic of China

## Abstract

**Objective:**

To investigate the differences in facial skin physiological parameters among women living in distinct ecological environments across five regions of Hainan Province, China.

**Methods:**

A total of 140 healthy adult women (28 per city) from Haikou, Qionghai, Dongfang, Wuzhishan, and Sanya were recruited. Facial skin parameters, including hydration, transepidermal water loss (TEWL), sebum, elasticity, pH, and colorimetric indices (L, a, b*, ITA°), were measured using a non‐invasive skin detector.

**Results:**

Higher concentrations of environmental pollutants (PM2.5, PM10, SO_2_, O_3_, CO, NO_2_) were significantly associated with increased TEWL, reduced skin hydration, and diminished elasticity (prolonged rebound time). Pollutants exhibited negative correlations with melanin, erythema, and the b* value (yellowness), but were positively correlated with brightness L* and ITA°.

**Conclusion:**

Urban differences in facial skin barrier function and color were observed. Air pollutant levels in a specific area were significantly associated with impaired skin barrier and altered skin color among local residents. This study explores the correlation between air pollution and skin aging in a tropical population.

## 1 Introduction

The skin serves as the first line of defense between the human body and the external environment. It protects the body from chemical and physical factors, prevents invasion by pathogenic microorganisms, and participates in metabolism, reabsorption, temperature regulation, and immune processes. The skin has a complex structure with unique epidermal barrier functions that maintain internal stability. The condition of the facial skin can reflect an individual's skin characteristics and health status [1, 2, 3, 4]. Non‐invasive skin function testing techniques, because of their convenience, speed, and non‐invasiveness, are increasingly being used to diagnose various skin diseases. These techniques primarily include dermatoscopy, high‐frequency ultrasound of the skin, confocal microscopy of the skin, and some new types of skin imaging equipment and software. Among these, the skin physiological function tester is a relatively new non‐invasive skin function testing technology that can assess the skin barrier by measuring transepidermal water loss (TEWL), epidermal pH, water content, and sebum content [5]. It can also evaluate skin tone through melanin, erythema, and skin brightness.

Nowadays, owing to multiple ecological issues such as vegetation destruction, industrial pollution emissions, vehicle exhaust emissions, and urban construction dust, the impact of pollutants in the air on facial skin is increasing day by day. A series of previous studies have identified exposure factors, including solar radiation, air pollution, tobacco smoke, and cosmetics, as key contributors to skin aging [6]. Air pollution mainly consists of two primary pollutants: particulate matter (PM), commonly referred to as fine particulate matter (elastic EPM2.5, PM10) or coarse particulate matter, and gases (ozone [O_3_], carbon dioxide [CO_2_], carbon monoxide [CO], sulfur dioxide [SO_2_], nitrogen dioxide [NO_2_]) or volatile organic compounds. Skin physiological parameters such as TEWL, skin elasticity VE, elastic E, recovery time, water content, oils, and PH value can reflect “skin barrier function,” while physiological indicators like melanin, erythema, skin brightness L*, a*, b* can reflect “skin tone.” Both aspects are closely related to skin aging. The relationship between air pollution and skin aging was first confirmed in the SALIA study, an epidemiological study of older white women. The study found that air pollution exposure is significantly associated with exogenous signs of skin aging, particularly pigmentation spots, indicating that exposure to traffic‐related particulates accelerates skin aging [7]. Subsequently, further cross‐sectional studies in China showed that using solid fuel for cooking is significantly associated with a 5%–8% increase in severe wrinkles on the face and a 74% increased risk of fine lines on the back of the hands, providing more epidemiological evidence linking exposure to fossil fuels with skin aging [8].

Hainan Province in China is located at the southernmost tip of the country and is the only tropical island province. Its geographical coordinates range from north latitude 18°10′ to 20°10′ and east longitude 108°37′ to 111°03′. The terrain of Hainan island is characterized by high central elevations (mountains and hills) and low, flat surroundings (plateaus). The central part features mountain ranges such as Wuzhishan and Limululing. Haikou City is situated in the northern part of Hainan Province; it has a tropical monsoon climate. The average annual temperature is approximately 24°C, and there are occasional damp and cold rainy days in winter. The annual precipitation ranges from 1600 to 1800 mm, and typhoons occur frequently with high humidity. Qionghai City is located on the eastern coast of Hainan Province. The Wanshui River alluvial plain makes it the center of heavy rainfall in eastern Hainan, with an annual precipitation exceeding 2000 mm. Typhoons often make landfall here, and winters are warmer and more humid than those in Haikou. Dongfang City is situated on the western leeward slope, influenced by the Foehn effect, with an annual precipitation of only 900–1200 mm, making it the driest region in the province. It enjoys ample sunshine; extreme summer temperatures can reach up to 38°C, with significant day‐night temperature differences. Wuzhishan City, owing to its higher altitude (300–1000 m) in the central mountainous area, has a cool mountain climate with an average annual temperature of 22°C–23°C and an annual precipitation of 1800–2400 mm. It experiences frequent fog and orographic rain, becoming a core area for escaping the heat in Hainan. Sanya City, located at the southernmost tip, is surrounded by mountains on three sides and faces the South China Sea to the south. It has no winter, with an average annual temperature of 25°C–28°C and an annual precipitation of 1200–1400 mm. Winters are dry and warm, with abundant sunlight. Overall, Hainan Island exhibits a “wet east, dry west; warm south, cool north” pattern [9, 10, 11].

In this study, healthy adult women living in Haikou, Qionghai, Dongfang, Wuzhishan, and Sanya were recruited and their physiological parameters, such as skin water content, TEWL, oil content, skin elasticity, PH value, and skin brightness, were collected to explore the influence of air pollution on the facial skin condition of women in tropical areas.

The measured physiological parameters in this study are well‐established biomarkers of skin aging. Elevated TEWL and decreased hydration are indicative of impaired stratum corneum function, which is a hallmark of aged skin. Reduction in skin elasticity, reflected by prolonged rebound time, is directly linked to the degradation of collagen and elastin in the dermis. Similarly, alterations in skin color parameters—such as melanin and erythema indices and the ITA° value—are associated with photoaging, which encompasses pigmentation disorders and compromised cutaneous microcirculation. Therefore, these non‐invasively measured parameters collectively provide a comprehensive assessment of skin aging manifestations. Hainan Island was selected as a representative tropical region for this study due to its unique ecological and climatic characteristics, which differ markedly from temperate or arid zones. Its location in the South China Sea results in a typical tropical monsoon climate with high annual temperatures, intense solar radiation, and high humidity levels. Furthermore, the island exhibits a distinct “wet east, dry west; warm south, cool north” climatic gradient. These conditions can modulate the effects of air pollutants on the skin; for instance, high humidity might enhance the adsorption of particulate matter onto the skin surface, while strong UV radiation can synergize with pollutants to induce oxidative stress. Studying skin aging in this under‐investigated tropical context provides valuable insights that may not be apparent in studies conducted in other climate zones.

## 2 Materials and Methods

### 2.1 Subjects

A total of 140 adult women (28 per city) were recruited for testing. The inclusion criteria were (1) Women who have lived in the area for more than 3 years; (2) Age between 18 and 50 years; (3) Not in their menstrual period; (4) Willing to cooperate with the survey; (5) Informed consent regarding the purpose and content of the study. The exclusion criteria were (1) Women who had undergone anti‐aging treatments such as phototherapy, injections, or chemical peels within the past 3 months; (2) Women recently exposed to or were about to be exposed to intense sunlight; (3) Women with comorbidities or tendencies towards anxiety, depression, schizophrenia, or other mental disorders; (4) Pregnant women; (5) Women with chronic conditions such as hypertension, diabetes, systemic lupus erythematosus, or allergic diseases such as solar dermatitis; (6) Women with facial acne, seborrheic dermatitis, melasma, or cafe‐au‐lait spots; (7) Women who had used oral or topical corticosteroids, immunosuppressants, antifungal medications, vitamin D3 derivatives, or retinoid drugs within the past month.

All recruited volunteers were urban residents with typical daily routines encompassing both indoor occupations and outdoor activities (e.g., commuting, shopping). While individual time‐activity patterns were not tracked, participants were all long‐term residents of their respective cities, implying sustained exposure to the general ambient pollution levels characteristic of each urban environment.

### 2.2 Measurement Conditions

The room temperature was maintained at 25°C ± 1°C, humidity at 80% ± 5%, and the room was supplied with natural light, avoiding direct sunlight. Test volunteers, as previously determined, cleaned their skin on the morning of the test day according to protocol requirements, assisted the tester in cleaning the test area, and gently wiped it clean with a dry paper towel. They then sat quietly for 30 min in an environment with constant temperature and humidity, during which they did not drink water or perform other activities, maintaining a relaxed state to ensure that the skin temperature was in sync with the room temperature.

### 2.3 Measuring Instruments and Methods

Skin analysis was performed using the DermaLab Combo System (Cortex Technology, Hadsund, Danish). The detection area was the left inner corner of the face, the outer corner of the eye, and the corner of the mouth. Each subject filled in a questionnaire on personal living habits and skin care habits. Questionnaire Data: The questionnaire was primarily used for screening purposes according to the strict inclusion and exclusion criteria (e.g., to identify recent sun exposure, cosmetic habits, or medical histories). These variables were not included as covariates in the final statistical models.

#### 2.3.1 Skin Water Content

The water test probe was applied vertically to the surface of the skin. The balance was maintained until the result was displayed on the computer. Each skin part was measured thrice; the average of the three measurements was used as the value (unit: C.U.).

#### 2.3.2 TEWL Value

The cylinder at the top of the probe was placed perpendicular to the skin surface. After the measurement starts, the instrument automatically collects TEWL value data every second, and the display screen displays these TEWL values as a curve, on which the average TEWL is also displayed. In this study, the value and deviation value were measured thrice, and the average of the three values was used. The unit was g/h m^2^. The lower the percutaneous water loss, the better the skin barrier function.

#### 2.3.3 Skin Oil Content

The skin oil tester is based on the principle of photometer and uses a special extinction tape (0.1 mm thick) to press on the test area for approximately 30 s. After it absorbs the oil on the skin, the tape becomes translucent, resulting in a change in light transmittance, which indirectly reflects the skin oil content (unit: μg sebum/cm^2^).

#### 2.3.4 Skin Chromaticity

The chromaticity system specified by the International Commission on Illumination (CIE) (Lab chromaticity system) is used to represent changes in skin tone. Here, L* represents luminance, and its variation indicates changes in the whiteness or blackness of the skin; the higher the value, the whiter the color tends to be. a* represents red‐green chromaticity, b* represents blue‐yellow chromaticity, and their combined evaluation can indicate the overall complexion.

The ITA° value is an individual type angle of the skin, representing the brightness of the skin in relation to L* and b*. The ITA° value indicates skin brightness; the higher the ITA° value, the brighter the skin. In this test, L* value, b* value, and ITA° value are used as parameters to evaluate skin color: ITA° = [Arctan (L* − 50/b*)] × 180/*π*.

#### 2.3.5 Skin Elasticity

Skin elasticity refers to the ability of skin to stretch and recover its original shape, which is usually related to the skin's collagen and elastin content. The average value was used after three tests. VE, E, and rebound time are all important parameters of elasticity.

#### 2.3.6 Ecological Environment Data

The daily average levels of PM2.5, PM10, SO_2_, O_3_, CO, and NO_2_ from September 1, 2024, to December 31, 2024, were collected through the open platform of the Hainan Provincial Department of Ecology and Environment. Daily averages were used directly without preprocessing.

### 2.4 Statistical Analysis

ANOVA and linear regression analysis were primarily used to analyze the data. First, descriptive analysis (Descriptive) was conducted on the data, followed by statistical analysis using SPSS Statistics 27.0 software (IBM Corporation, Armonk, NY, USA). One‐way ANOVA (ANOVA) and pairwise comparisons were used to determine if the differences in skin parameters and environmental pollutants among different cities were statistically significant. Statistical tests were performed with two‐tailed methods, with a significance level of 0.05 (*p* < 0.05). Linear regression analysis was employed to demonstrate the correlation between environmental pollutant data and skin physiological indicators. Sample Size Consideration: The sample size of 28 participants per city was determined based on logistical feasibility and participant accessibility within the study period. While this provides initial insights, we acknowledge that it may limit the statistical power to detect smaller effects, and the findings should be interpreted as exploratory.

## 3 Results

**FIGURE 1:**
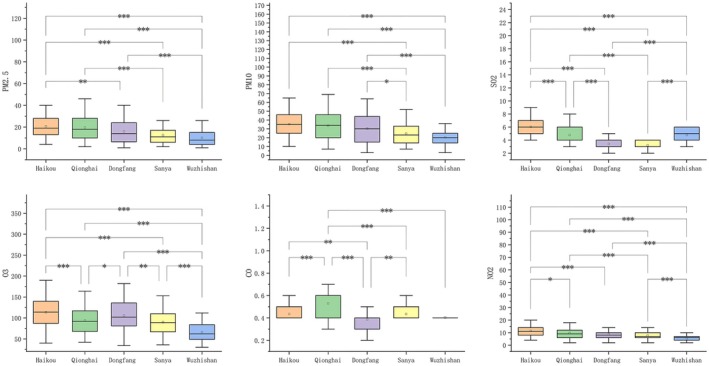
Comparison chart of air pollutant indicators across five cities. PM2.5 (A), PM10 (B), SO_2_ (C), O_3_ (D), CO (E), NO_2_ (F). * indicates a significant difference between the two cities (*p ≤ 0.05, **p ≤ 0.01, ***p ≤ 0.001).

The PM2.5 and PM10 concentrations in Haikou and Qionghai were significantly higher than those in Dongfang, Sanya, and Wuzhishan. There was no statistically significant difference between Haikou and Qionghai. Dongfang had higher concentrations than Sanya, while Wuzhishan had the lowest PM2.5 and PM10 concentrations (Figure 1A,B). The SO_2_ concentration in Haikou was significantly higher than in the other four cities, with no statistically significant difference between Dongfang and Sanya or Qionghai and Wuzhishan. The O_3_ concentration in Haikou and Dongfang was higher than in Qionghai, Sanya, and Wuzhishan (Figure 1C). There was no statistically significant difference between Qionghai and Sanya, but the O_3_ concentration in Wuzhishan was the lowest and the difference was statistically significant (Figure 1D). The CO concentration in Qionghai was the highest; the CO concentrations in Haikou and Sanya were significantly higher than those in Dongfang and Wuzhishan, with a statistically significant difference (Figure 1E). The NO_2_ concentration in Haikou was the highest, followed by Qionghai, with no significant difference between Dongfang and Sanya and the lowest concentration was seen in Wuzhishan (Figure 1F).

**FIGURE 2:**
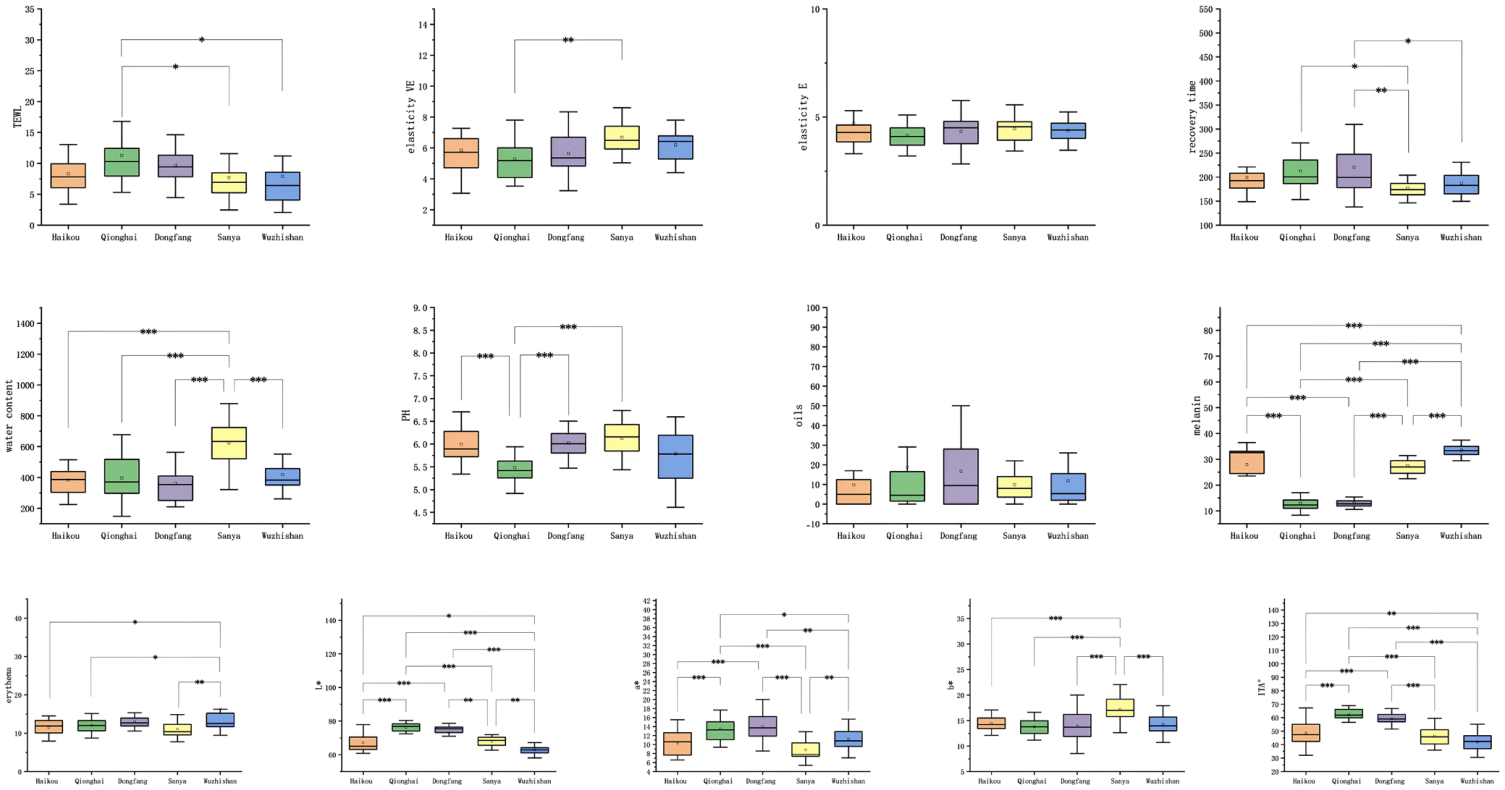
Comparison chart of air pollutant indicators across five citie. TEWL (A), elasticity VE (B), elasticity E (C), recovery time (D), water content (E), PH (F), oils (G), melanin (H), erythema (I), L*(J), a*(K), b*(L), ITA (M). Angle ITA (e); skin yellowness (b*) value (f); skin melanin index value (g); skin erythema index value (h). * indicates a significant difference between the two cities (*p ≤ 0.05, **p ≤ 0.01, ***p ≤ 0.001).

The TEWL of facial skin was most significant in Qionghai, followed by Dongfang, with no noticeable differences among Haikou, Sanya, and Wuzhishan (Figure 2A); only Qionghai and Sanya exhibited significant differences in the elasticity VE indicators (Figure 2B); there were no statistically significant differences in the elasticity E and sebum levels of facial skin across regions (Figure 2C,G). The recovery time for elasticity showed a statistically significant difference between Dongfang and Sanya, with Dongfang having a longer recovery time (Figure 2D). The skin hydration levels were highest in the Sanya region, with no statistically significant differences among the other four cities (Figure 2E). The PH values showed a significant difference between Haikou and Qionghai, while there were no statistically significant differences in the other cities (Figure 2F). The melanin levels were highest in Wuzhishan, followed by Haikou and Sanya, with the lowest melanin levels found in Qionghai and Dongfang (Figure 2H). The number of erythema spots on the skin was significantly different between Wuzhishan and other cities (Figure 2I). The L* and ITA°values were the highest for participants from Qionghai, indicating the whitest complexion, while those from Wuzhishan had a darker complexion (Figure 2J,M). The facial a* values were higher in Qionghai and Dongfang, with a redder appearance (Figure 2K). The b* value was the highest for participants from Sanya, indicating a more yellowish complexion (Figure 2L).

The atmospheric CO level is positively correlated with TEWL; PM2.5 and PM10 are negatively correlated with elastic VE, while PM2.5, PM10, O_3_, and NO_2_ are positively correlated with rebound time. The PM2.5, PM10, SO_2_, and NO_2_ are all negatively correlated with the skin's water content; pH value and oils have no correlation with environmental pollutants. The melanin level is negatively correlated with all pollutants; skin erythema is negatively correlated with PM2.5, PM10, O_3_, and NO_2_. The skin brightness‐L* is positively correlated with all pollutants except SO_2_; brightness‐a* is positively correlated with PM2.5 and PM10; brightness‐b* is negatively correlated with PM2.5, PM10, and SO_2_. The skin type angle (ITA) is positively correlated with PM2.5, PM10, CO, O_3_, and NO_2_ (Table 1).

**TABLE 1:** Linear regression analysis of the correlation between air pollutant data and skin physiological parameter data.

	PM2.5	PM10	SO_2_	O_3_	CO	NO_2_
TEWL	0.174 [−0.011, 0.36] *p* = 0.065	0.126 [−0.009, 0.261] *p* = 0.67	0.58 [−0.677, 0.793] *p* = 0.876	0.018 [−0.028, 0.065] *p* = 0.444	**16.584 [1.494, 31.674]** ***p* = 0.031**	0.233 [−0.147, 0.613] *p* = 0.228
Elasticity‐VE	**−0.083 [−0.146**, −**0.019]** ***p* = 0.011**	**−0.059 [−0.105**, −**0.013]** ***p* = 0.012**	−0.173 [−0.425, 0.078] *p* = 0.179	−0.011 [−0.027, 0.005] *p* = 0.163	−4.401 [−9.635, 0.834] *p* = 0.099	−0.126 [−0.255, 0.004] *p* = 0.058
Easticity‐lE	0.008 [−0.044, 0.061] *p* = 0.758	0.006 [−0.032, 0.044] *p* = 0.754	0.075 [−0.130, 0.280] *p* = 0.469	0.005 [−0.008, 0.018] *p* = 0.459	−1.492 [−5.769, 2.785] *p* = 0.492	0.034 [−0.072, 0.141] *p* = 0.524
Recovery time	**2.443 [0.621, 4.264]** ***p* = 0.009**	**1.897 [0.575, 3.219]** ***p* = 0.005**	1.304 [−6.006, 8.613] *p* = 0.725	**0.526 [0.071, 0.981]** ***p* = 0.024**	53.200 [−99.196, 205.596] *p* = 0.491	**3.862 [0.119, 7.604]** ***p* = 0.043**
Hydration	**−11.052 [−17.267**, −**4.837]** ***p* < 0.001**	**−7.972 [−12.503**, −**3.442]** ***p* < 0.001**	**−49.508 [−73.516**, −**25.500]** ***p* < 0.001**	−1.529 [−3.118, 0.061] *p* = 0.059	86.124 [−443.990, 616.239] *p* = 0.749	**−15.391 [−28.331**, −**2.450]** ***p* = 0.020**
pH value	0.024 [−0.039, 0.088] *p* = 0.450	0.020 [−0.027, 0.066] *p* = 0.401	0.096 [−0.153, 0.345] *p* = 0.448	0.014 [−0.002, 0.029] *p* = 0.089	−3.082 [−8.265, 2.101] *p* = 0.242	0.085 [−0.044, 0.214] *p* = 0.193
Sebum	0.253 [−0.578, 1.084] *p* = 0.548	0.194 [−0.411, 0.800] *p* = 0.527	−0.597 [−3.856, 2.661] *p* = 0.718	0.011 [−0.196, 0.217] *p* = 0.918	29.388 [−38.496, 97.272] *p* = 0.394	0.147 [−1.546, 1.841] *p* = 0.864
Melanin	**−1.131 [−1.483**, −**0.778]** ***p* < 0.001**	**−0.902 [−1.151**, −**0.653]** ***p* < 0.001**	**2.351 [0.834, 3.868]** ***p* = 0.003**	**−0.261 [−0.350**, −**0.172]** ***p* < 0.001**	**−69.468 [−100.044**, −**38.893]** ***p* < 0.001**	**−1.820 [−2.575**, −**1.065]** ***p* < 0.001**
Erythema	**−0.285 [−0.520**, −**0.050]** ***p* = 0.018**	**−0.217 [−0.388**, −**0.046]** ***p* = 0.013**	0.162 [−0.778, 1.102] *p* = 0.733	**−0.085 [−0.143**, −**0.028]** ***p* = 0.004**	−17.533 [−36.941, 1.876] *p* = 0.076	−**0.687 [−1.162**, −**0.212]** ***p* = 0.005**
Brightness‐L*	**0.709 [0.445, 0.972]** ***p* < 0.001**	**0.545 [0.355, 0.735]** ***p* < 0.001**	−1.026 [−2.145, 0.093] *p* = 0.072	**0.146 [0.078, 0.213]** ***p* < 0.001**	**57.077 [35.467, 78.687]** ***p* < 0.001**	**1.201 [0.648, 1.753]** ***p* < 0.001**
Brightness‐a*	**0.156 [0.025, 0.286]** ***p* = 0.020**	**0.123 [0.029, 0.218]** ***p* = 0.011**	−0.085 [−0.607, 0.436] *p* = 0.746	0.020 [−0.012, 0.053] *p* = 0.221	5.640 [−5.202, 16.481] *p* = 0.305	0.134 [−0.135, 0.404] *p* = 0.326
Brightness‐b*	**−0.139 [−0.253**, −**0.025]** ***p* = 0.017**	**−0.098 [−0.181**, −**0.015]** ***p* = 0.021**	**−0.613 [−1.056**, −**0.170]** ***p* = 0.007**	−0.011 [−0.040, 0.017] *p* = 0.432	−2.822 [12.297, 6.654] *p* = 0.577	−0.166 [−0.401, 0.068] *p* = 0.162
ITA°	**1.253 [0.872, 1.634]** ***p* < 0.001**	**0.964 [0.691, 1.236]** ***p* < 0.001**	−0.991 [−2.686, 0.705] *p* = 0.250	**0.256 [0.157, 0.355]** ***p* < 0.001**	**80.611 [47.712, 113.510]** ***p* < 0.001**	**2.065 [1.250, 2.879]** ***p* < 0.001**

*Note:* Bold, significant associations.

In summary, in our study, the higher the concentrations of environmental pollutants such as PM2.5, PM10, SO_2_, O_3_, CO, and NO_2_, the greater the TEWL, the poorer the skin hydration (i.e., the skin's ability to retain moisture), and the more reduced the elasticity (increased rebound time). The skin barrier function was also found to be impaired. Air pollutants were found to be negatively correlated with skin pigmentation indicators such as melanin, erythema, and skin brightness b*, but positively correlated with brightness L* and ITA.

## 4 Discussion

Analysis of the five cities revealed a clear pollution gradient. Haikou, the economic center, suffered the most severe pollution due to intensive traffic and industry. In contrast, Wuzhishan's pristine mountainous environment with high forest cover resulted in the cleanest air. The other cities exhibited intermediate and distinct profiles: Qionghai's agricultural setting faced compound pollution from biomass burning and sea spray; Dongfang's industrial activity led to high O_3_, though its dry climate limited PM buildup; and Sanya's tourism‐driven emissions were partially offset by coastal winds that dispersed particulate matter.

Particulate matter (PM), especially fine particles like PM2.5 (diameter ≤ 2.5 μm) and PM10 (diameter ≤ 10 μm), is a major harmful air pollutant. Due to their small size—smaller than skin pores—these particles can penetrate the skin through hair follicles [12, 13]. PM2.5 can even be inhaled into the alveoli, affecting lung function [14]. Once inside, they trigger two key damaging mechanisms. First, components adsorbed onto PM activate the aryl hydrocarbon receptor (AhR), a transcription factor expressed in skin cells that regulates skin barrier function and homeostasis [15, 16, 17, 18]. Second, PM induces oxidative stress by generating reactive oxygen species (ROS) [19]. This elevated ROS level leads to collagen degradation, damages the extracellular matrix, and disrupts skin barrier function, resulting in clinical signs such as dryness, decreased elasticity, and accelerated skin aging [20, 21, 22, 23], and even increasing the risk of skin cancer [24]. The findings from Ding et al., which linked PM2.5 exposure to skin aging in a Chinese population [25], align with our observed results of reduced skin hydration and prolonged rebound time. Our findings that PM2.5 and PM10 were associated with decreased skin hydration and impaired elasticity are consistent with previous studies conducted in China and Germany [7, 25]. For instance, Ding et al. also reported that indoor PM2.5 exposure was linked to compromised skin barrier function in a Chinese population [25]. The proposed mechanisms, including oxidative stress and AhR activation, align with the experimental work of Dijkhoff et al. [23].

O_3_ present in the atmosphere can absorb ultraviolet light, protecting living organisms on Earth from excessive ultraviolet damage. In the environment, short‐term exposure to O_3_ is beneficial to human skin, as it increases oxygen supply and accelerates wound healing. However, long‐term exposure can have many adverse effects on human skin, such as oxidative degradation of biomolecules and the production of reactive oxygen species, leading to impaired skin barrier function, loss of collagen, and cellular toxicity [26, 27, 28]. The 2019 study by Fuks et al. found that higher levels of O_3_ exposure result in more forehead wrinkles [29] Our research also shows a positive correlation between O_3_ concentration and skin recovery time, indicating that increased O_3_ concentration leads to decreased skin elasticity after collagen loss. Long‐term exposure to high concentrations of O_3_ depletes antioxidants in the stratum corneum [30]. O_3_ also increases lipid peroxidation and protein oxidation in mouse skin [31, 32]. However, some studies have found that O_3_ levels are negatively correlated with skin texture and pore size, possibly because of lower O_3_ concentrations at the study sites [33]. The positive correlation between O_3_ concentration and skin recovery time (indicating reduced elasticity) observed in our study supports the findings of Fuks et al., who reported an association between tropospheric ozone and increased forehead wrinkles in German cohorts [29]. This suggests that ozone‐induced collagen degradation might be a common pathway in different populations.

Nitrogen dioxide (NO_2_) has been shown in multiple studies to increase TEWL and impair skin barrier function [34, 35, 36]. In our study, while O_3_ was not significantly correlated with TEWL, it was negatively associated with skin rebound time—a discrepancy that may stem from limited sample size, regional variations in pollutant concentrations, or differences in personal protection habits. Region‐specific mechanisms may further explain skin damage: in Qionghai, high PM2.5 levels combined with humid conditions may enhance particle adsorption onto the skin, disrupting stratum corneum lipids and worsening water loss. In contrast, Dongfang's lower PM but high O_3_ exposure likely accelerates collagen degradation via oxidative stress, leading to impaired elasticity and prolonged rebound time.

Beyond accelerating aging, air pollutants are implicated in several common skin diseases. They may independently trigger or worsen atopic dermatitis (AD) in both children and adults [37, 38, 39]. Prenatal exposure to PM and NO_2_, particularly in early pregnancy, is also associated with an increased risk of AD in offspring [40, 41, 42]. Furthermore, PM [43] and O_3_ [44] can promote Th17 differentiation through AhR‐dependent pathways, potentially exacerbating psoriasis. In a Chinese cohort, exposure to PM and NO_2_ was linked to more severe acne lesions and elevated sebum production [45], correlating with higher clinic visitation rates [46]. Interestingly, the negative correlations between air pollutants and melanin/erythema in our study contrast with some previous reports that linked NO_2_ exposure to increased lentigines in older Caucasian and Chinese women [7, 47]. However, our results are more aligned with a recent study in Taiwan by Chao‐Hsin H et al. [33], which also found negative correlations between PM10, PM2.5, SO_2_, CO, and pigmentation. This discrepancy highlights the potential influence of geographic, climatic, ethnic, and age differences, suggesting that the relationship is not uniform and warrants further investigation.

It is important to distinguish between overall skin color and specific pigmented spots, although both are influenced by environmental stressors and are relevant to skin aging. Our study focused on general skin color parameters (L, a, b, ITA°), which reflect the cumulative impact of environmental exposures on skin pigmentation and vascularization. Chronic exposure to air pollutants and UV radiation can lead to oxidative stress and inflammation, contributing to uneven skin tone, sallowness (decreased b), and the formation of lentigines—all of which are clinical features of extrinsic skin aging. While we did not quantitatively assess discrete spots, the observed negative correlation between pollutants and melanin/erythema might suggest a complex interplay where chronic exposure in some tropical contexts could initially suppress some inflammatory and pigmentation markers, contrary to findings focused solely on spot count in older cohorts. The relationship between air pollution and skin pigmentation is likely non‐linear and dependent on factors such as exposure duration, pollutant mixture, and individual susceptibility.

This study has several limitations that should be considered. First, its cross‐sectional design allows only for the description of associations and cannot establish causality between air pollution and skin changes. Second, the modest sample size may affect the power to associations. Third, we collected the daily average level of air pollutants from September 1 to December 31, 2024. We noticed that this 4‐month period does not represent the annual change of pollutants and may not take into account the potential seasonal impact on skin parameters. Fourthly, our statistical models did not adjust for potential confounders such as age, skin phototype, individual skincare routines, dietary habits, or socioeconomic status, which may influence skin physiology. Future longitudinal studies with larger sample sizes and more comprehensive data collection are needed to confirm these findings and elucidate the underlying causal mechanisms. Additionally, the environmental data were collected over a four‐month period, which does not capture seasonal variations in pollution and climate that could impact skin parameters.

## 5 Conclusion

This study demonstrates distinct urban variations in facial skin barrier characteristics and skin color among women residing in different ecological environments of Hainan Province. Higher concentrations of ambient air pollutants were significantly associated with impaired skin barrier function, evidenced by increased TEWL, decreased hydration, and reduced elasticity. Furthermore, specific air pollutants showed significant correlations with skin color parameters: negative correlations with melanin, erythema, and the yellow‐blue component (b), and positive correlations with skin lightness (L) and ITA°. These alterations in both skin barrier integrity and pigmentation are recognized manifestations of extrinsic skin aging. Therefore, our findings suggest that chronic exposure to region‐specific air pollution in this tropical setting contributes to the accelerated aging of facial skin. It is crucial to emphasize that these findings, derived from a cross‐sectional study, indicate association and not causation. Future studies incorporating longitudinal designs, personal exposure monitoring, and assessment of specific aging signs like wrinkles and spots are warranted to further elucidate these complex relationships.

## Author Contributions

Jingqiu Fu and Wenxia Huang: investigation, data curation, formal analysis, Writing – original draft. Yujia Liang: investigation. Rui Wang and Xiaoli Chen: data curation. Jiejie Lu: resources, conceptualization. Weiwei Wu: funding acquisition, project administration, supervision, writing – review and editing, conceptualization.

## Funding

This work was supported by the Construction Project of Hainan Province Clinical Medical Center (No. QRCBT202121).

## Ethics Statement

The studies involving humans were approved by The Fifth People's Hospital of Hainan (number: 2024‐13). The studies were conducted in accordance with the local legislation and institutional requirements. The participants provided their written informed consent to participate in this study.

## Conflicts of Interest

The authors declare no conflicts of interest.

## Data Availability

The data that support the findings of this study are available from the corresponding author upon reasonable request.
